# Validity and Reliability of the Korean Version of the Anesthesia Surrendering Instrument

**DOI:** 10.3390/ijerph17093065

**Published:** 2020-04-28

**Authors:** Hanna Lee, Ji-Soon Kang, Jeong-Won Han

**Affiliations:** 1 Department of Nursing, Gangneung-Wonju National University, Wonju-si 26403, Korea; 2 Department of Nursing, Hansei University, Gunpo-si 43742, Korea; 3 College of Nursing Science, Kyung Hee University, Seoul 02447, Korea

**Keywords:** anesthesia, patients, reproducibility of results

## Abstract

This study examined the reliability and validity of the Korean version of the anesthesia surrendering instrument (ASI), which was originally developed to measure anesthesia surrendering in Swedish adults. The study population consisted of 306 patients who received general anesthesia for abdominal, breast, knee, hip, lower back, or shoulder surgery in ten hospitals across five regions of Korea from June to September 2019. The validity of the content, construct, and criterion used, and the reliability of the ASI were assessed. The results showed that the instrument had appropriate content validity; the item-level content validity index ranged between 0.80 and 1.00, and the scale-level content validity index was 0.90. The construct validity test results confirmed four sub-categories with a total of 26 items, and the internal consistency reliability tests showed Cronbach’s alpha values ranging between 0.71 and 0.88. The study findings confirmed the applicability of this instrument for measuring anesthesia surrendering in Korean adults. These results provide a foundation for future studies on anesthesia surrendering in Korean adult patients.

## 1. Introduction

South Korea has a National Health Insurance (NHI) system and advanced medical technology, and there has been a significant increase in the number of individuals visiting medical institutions to maintain and improve their health [1]. NHI system in South Korea is compulsory, required by Korean law, and everyone resident in the country is eligible regardless of nationality or profession [2]. The system is funded by compulsory contributions from all residents and government subsidies. Payment is made by an individual’s employer unless they are self-employed, in which case they pay it themselves [3]. Foreigners living in South Korea who are registered with the National Health Insurance Corporation (NHIC) receive the same medical benefits and services as Korean nationals [3].

According to the NHIC, the total medical expenditure in Korea in 2016 was 6.0 billion dollars, wherein surgical costs accounted for 68.0% at 4.1 billion dollars in a complete enumeration [4]. The number of surgical cases also increased 1.3-fold from 1.371 million in 2006 to 1.765 million in 2017 [5]. Anesthesia is broadly classified as general and regional [6]. General anesthesia refers to the loss of consciousness accompanied by suppression of sensory, motor, and reflex stimulation; patients consider surgery requiring general anesthesia as a major invasive procedure [7]. Patients requiring general anesthesia for surgery are generally apprehensive; they become anxious about the outcomes and recovery of consciousness [8]. Moreover, patients who exhibit high levels of anxiety frequently distrust medical personnel who administer anesthesia [9] and show low levels of compliance during anesthesia induction [10]. Additionally, preoperative stress associated with anesthesia may stimulate the sympathetic nervous system to up-regulate the secretion of norepinephrine and epinephrine, as well as increase other physiological responses such as cardiac output, blood sugar level, blood pressure, bronchial dilation, contraction of the peripheral vessels, and muscle tension, which lead to complications during surgery or postoperative recovery [11].

The medical environment in South Korea requires nurse to provide professional nursing during the operation time of the patient [12]. So anesthetic nurses in Korea cooperate with other medical staff and perform various nursing activity such as identification of subjects before surgery, anesthesia during surgery, pain management, monitoring of cardiopulmonary condition and emergency care. [13]. The effectiveness of nursing interventions during the administration of anesthesia depends on the dynamic, personal, and therapeutic relationship formed between the patient and the nurse during the induction of anesthesia [8,14]. It is imperative that nurses not only safely administer anesthesia and maintain a deep sleep state in patients until the completion of the surgery, but also ensure that patients fully understand its implications and safely surrender to its induction [11,14]. In general, surrender can be defined as a psychological state that allows an individual to entrust their safety to another person. Anesthesia surrendering in patients is the surrendering of one’s physical safety and health to the care of medical personnel [8]. Surrendering implies that an individual is assured of their safety, and it enables genuine psychological relaxation. The ease of anesthesia surrendering depends on the patients’ interactions with medical personnel [8,14,15]. Therefore, medical personnel should facilitate anesthesia induction by addressing the concerns of patients so that they can voluntarily surrender to the process [8].

To alleviate patients’ anxiety related to the administration of anesthesia and to increase compliance, nurses need a thorough understanding of anesthesia surrendering and a means of quantifying the experience. Therefore, there is a need for appropriate assessment tools that can help medical personnel in providing necessary interventions during general anesthesia induction. The anesthesia surrendering instrument (ASI) is one such tool that can be used to assess anesthesia surrendering in patients before surgery [16]. It comprises 27 items related to the anesthesia surrendering experience, as studied in Swedish adults. These items are classified under four dimensions of the surrendering experience: preparation by avoidance, control, preparation by understanding, and acceptance. The instrument has been previously tested and found to be suitable for measuring the experience of anesthesia surrendering in patients. In South Korea, the number of doctors per capita is 16.6, the highest among OECD (Organization for Economic Cooperation and Development) countries, and patients are provided with the conditions to see medical treatment whenever they want [17]. However, it has been reported that patients in South Korean complain of extreme anxiety if they do not receive sufficient information or intervention on treatment directly related to their lives such as surgery [18].

Since there is a need to reduce surgical complications and measure the performance of interventions for general anesthesia surrendering, the present study aimed to translate the ASI, originally developed by Liebenhagen and colleagues [16], into a tool that could be used for Korean patients and test its validity and reliability.

## 2. Materials and Methods

### 2.1. Subjects

The study population consisted of patients who received general anesthesia for abdominal, breast, knee, hip, lower back, or shoulder surgery. All participants were above 18 years of age and provided informed consent after understanding the study protocols. They had no difficulty in reading and understanding the materials provided in Korean. Patients who had undergone surgery for cancer in qualifying areas were excluded from the study since their anxiety could differ from that of other surgery patients because of their cancer diagnosis. The number of hospital beds was used as a stratification variable for the study sample and based on a previous study [19], which used Dalenius & Hodges’s cumulative-square root method, the following strata were determined: 100–299, 300–499, 500–999, and more than 1000 beds. Hospitals with more than 1000 beds were excluded from the sample. Based on a previous study [20], hospitals between 200 and 300 beds were included. Additionally, five of the top ten regions were selected from the list of 162 demographic cities, using the Republic of Korea National Statistical Portal [21]. Finally, the participants were administered anesthetic agents by specialists in medical institutions with capacities of 200 beds or more. Patients with similar durations of anesthesia induction were selected as participants based on the discussions with medical personnel at each medical institution and the previous study [16]. To perform exploratory factor analysis (EFA), an items-to-sample-size ratio of 1:5 or at least 100 respondents were required [22]; a sample size of at least 150–200 is appropriate for confirmatory factor analysis (CFA) [23]. The minimum sample needed for this study was estimated to be 300 subjects. A total of 306 participants were selected (retrieval rate, 95.9%), which satisfied the aforementioned sample-size requirement. The questionnaires completed by the selected participants were used for preliminary (EFA, *n* = 100) and secondary factor structure testing (CFA, *n* = 206).

### 2.2. Study Tools

#### 2.2.1. Anesthesia Surrendering Instrument

The ASI was developed by Liebenhagen and colleagues [16] and comprises 27 items classified under four dimensions; 7, 7, 7, and 6 items for the preparation by avoidance, control, preparation by understanding, and acceptance dimensions, respectively. Each item was scored on a 4-point scale (1 point for “highly disagree” to 4 points for “highly agree”) and higher scores indicated higher levels of anesthesia surrendering. At the time of development, the reliability of the instrument, as indicated by Cronbach’s alpha, was 0.76.

#### 2.2.2. State-Trait Anxiety Inventory

We used the State-Trait Anxiety Inventory (STAI) form-Korean YZ (STAI-KYZ), which is a self-reported STAI developed by Spielberger [24] and adapted for Korean adults by Han et al. [25]. In the present study, only 20 items related to state anxiety were used. Each item was scored on a 4-point Likert scale, with higher scores indicating a higher level of anxiety. The reliability of the instrument, as measured by Cronbach’s alpha, was 0.92 in the study by Han et al. [25] and 0.87 in our study.

### 2.3. Data Collection

Data were collected for the period between 1 June 2019, and 30 September 2019, from five regions in Korea (“S” city, “G” do, and “I,” “B,” and “C” metropolitan cities), including major and small-to-medium sized cities. Ten hospitals within these regions were convenience-sampled, and the authors interacted with the nurses and various department personnel before data collection to explain the study protocols. Informed written consent was obtained from patients with the cooperation of relevant department heads. For patients who were scheduled to be discharged after surgery, the researchers made an effort to sufficiently explain the purpose and methods of the study on the day of, or the day before, discharge and obtained informed written consent from those who wanted to participate.

### 2.4. Study Procedure

The present study followed the guidelines for the translation and adaptation of English language instruments for use in other languages, as recommended by the World Health Organization [26]. The finalization of the items used in the instrument involved a process of preliminary translation, expert panel review, back translation, and cognitive assessment. Before the preliminary translation step, approval for the translation into Korean and its subsequent use was obtained from the original developer of the instrument. A healthcare professional whose native language was Korean, but was familiar with the technical terminology of the relevant field and well-versed in English, was best qualified to perform the preliminary translation. Consequently, two Doctors of Nursing Practice (DNPs) who were fluent in English and had sufficient experience with technical terminology in the nursing field were assigned to perform the preliminary translation. A consensus was reached on the translated content following the independent preliminary translations by the two DNPs, to ensure that literal translation was avoided and the intended meaning of each sentence, not individual words, was accurately conveyed. Subsequently, an expert panel, including a professional bilingual translator, two nursing professors fluent in both languages, and the two DNPs who performed the preliminary translation, was established and the translated sentences were reviewed through comparison with the original English version. The consistency and accuracy of the translated sentences were verified and, based on considerations of readability and cultural differences, some portions were revised. An American whose native language was English, but was fluent in Korean, and was not familiar with the instrument was assigned to perform the back translation. Although the back-translated content did not perfectly match the content in the original instrument, it was confirmed that there were no changes in the intended meaning. The aforementioned rigorous translation process ensured content validity [26]. Content validity was also verified by an anesthesiologist, a DNP with over 10 years of clinical nursing experience, two nursing professors, and a Ph.D. in nursing student. The appropriateness, sufficiency, and representativeness of the items in each dimension were examined using the item-level content validity index (I-CVI) and the scale-level content validity index/AVE (S-CVI/AVE). The S-CVI was calculated as the average of the I-CVI by experts for content validity analysis. The CVI is a 4-point scale that was reported by Polit et al. [27]. The I-CVI estimates appropriate content validity at 0.80 or higher and when the S-CVI is 0.90 [27]. This study additionally confirmed content validity through cognitive validity testing with three people who had had similar surgery experiences as the patients the instrument would be used for. The participants were asked to read the items of the tool aloud one at a time, and the readability and comprehension of the items were expressed in words.

### 2.5. Data Analysis

Data were analyzed using AMOS (version 22.0; IBM, Armonk, NY) and SPSS (version 20; IBM). The frequency, percentage, mean, and standard deviation (SD) were calculated for the general characteristics of participants. Skewness and kurtosis were estimated to confirm the normality of the data using item analysis and verified them by the multivariate normality test using AMOS. In this study, EFA and CFA were conducted to verify construct validity, and different subjects were required for each of them [28]. Therefore, data for 100 subjects were randomly selected from the collected data for EFA, using Microsoft Excel (Microsoft; Redmond, WA), and the remaining data were used for CFA. For EFA, the principal axis factor analysis was used for extraction in the common factor model and the varimax rotation, considering the correlations between factors. The varimax rotation was applied to minimize the number of factors and information loss, and the Kaiser-Meyer-Olkin (KMO) test and Bartlett’s test of sphericity were performed to confirm the suitability of the data for factor analysis. If the KMO test result was greater than 0.50, the data were found to be suitable for analysis [28]. Several methods are suggested for determining the number of factors, such as classification of more than one eigenvalue (commonly used), parallel analysis, and Velicer’s minimum average partial analysis [29,30]. All three aforementioned methods were used in this study. The number of factors was determined using eigenvalues of 1.0 or higher, and items were selected based on a factor loading of 0.40 or higher. The Weighted Least Square Mean And Variance (WLSMV), calculated using Mplus (Los Angeles, CA), was applied to estimates that did not satisfy multivariate normality, and the Likert scales for the CFA were considered for these [31]. The goodness-of-fit index was verified by χ^2^ values (degree of freedom or df, *p*-value), weighted root mean square residuals (WRMR; reference value: ≤ 1.0), the Tucker-Lewis index (TLI; reference value: ≥ 0.9), the comparative fit index (CFI; reference value: ≥ 0.9), and the root mean square error of approximation (RMSEA; reference value: ≤ 0.05). The convergent validity of the factor construct was based on the following criteria: standardized regression coefficients of observed variables ≥ 0.50, construct reliability (CR) ≥ 0.7, and average variance extracted (AVE) ≥ 0.5. The discriminant validity of the factors estimated by CFA is verified if the inter-factor correlation coefficients are ≤ 0.80 and the AVE of latent variables is greater than the square of correlation coefficients of the latent variables. To test the criterion validity of the Korean ASI, a gold standard scale with proven validity and reliability was required. However, because there are very few instruments designed to measure anesthesia surrendering and almost none have proven validity and reliability, the findings from this study were compared to a previous study [7] on anxiety in general anesthesia patients. Hence, the Pearson’s correlation coefficient between the Korean ASI and STAI-KYZ was obtained for criterion validity. To measure internal consistency, Cronbach’s alpha correlation coefficient was obtained.

### 2.6. Ethical Consideration

This work was approved by the Institutional Review Board of Changwon University (1040271-201902-HR-002).

## 3. Results

### 3.1. General Characteristics of Subjects

The study population included 88 males (28.7%) and 218 females (71.3%), with a mean age of 45.91 ± 16.60 years. Of them, 202 individuals (66.0%) were married, 92 (30.1%) were unmarried, and 12 (3.9%) were widowed or divorced. Additionally, 260 (84.9%) were employed and 46 (15.1%) were unemployed. The sites of surgery included the abdomen (*n* = 126, 41.2%), breast or ovary (*n* = 60, 19.6%), knee (*n* = 32, 10.5%), hip (*n* = 32, 10.5%), lower back (*n* = 36, 11.7%), and shoulder (*n* = 20, 6.5%).

### 3.2. Validity Analysis

#### 3.2.1. Content Validity

Almost all items (26/27) showed an I-CVI ranging between 0.80 and 1.00 and S-CVI/AVE of 0.90, indicating adequate content validity. The item “I looked around the operating theater” had an I-CVI of 0.80 and S-CVI/AVE of ≤ 0.90. However, it was determined that this was a coping behavior in patients during anesthesia surrendering, and consequently, all 27 items were retained.

#### 3.2.2. Item Analysis

We analyzed the skewness and kurtosis to ascertain the normality of the data used in this study. The skewness of all measured variables was between 0.01–1.15 (absolute value) and did not exceed 3; kurtosis did not exceed 10 and had absolute values between 0.04–1.41. The data showed normal distribution. However, the multivariate normality test showed that the multivariate kurtosis was 43.5 and the critical ratio was 14.2 (Table 1). It is not practical to confirm the combined frequency distributions for all variables, and few data satisfy the assumption of multivariate normality. Therefore, if there is no major problem with a univariate normality test, it can be assumed that multivariate normality is satisfied [32]. In summary, the measured variables in this study satisfied univariate normality, and thus, the validity of the ASI was satisfactory.

#### 3.2.3. Construct Validity

This study determined the number of factors through the classification of more than one eigenvalue, parallel analysis, and Velicer’s minimum average partial analysis. Four factors were found using eigenvalues, two factors using parallel analysis, and two factors using Velicer’s minimum average partial analysis. Based on the number of factors used for the original ASI, four factors for the factor analysis were finalized. EFA was performed for all 27 items, applying the principal component analysis and varimax rotation. The KMO value for determining the suitability of samples for factor analysis was 0.85, which was higher than the cutoff value of 0.80. The approximate χ^2^ value for Bartlett’s test of sphericity was 2942.88 (df = 231, *p* < 0.001), which indicated that the samples were suitable for factor analysis. Factor analysis of all 27 items showed four factors with an initial eigenvalue ≥ 1.0. The item, “I looked around the operating theater”, was excluded for its commonality of 0.10; the other items had commonalities ≥ 0.50 and satisfied the factor loading criterion of ≥ 0.40. Based on the EFA results, 26 out of the 27 initial items were retained (Table 2).

#### 3.2.4. Confirmatory Factor Analysis

CFA was performed for 26 items under the four factors identified by preliminary construct validity testing. The goodness-of-fit assessment showed χ^2^ = 1022.33 (df = 293, *p* < 0.001), WRMR = 1.05, TLI = 0.92, CFI = 0.93, and RMSEA = 0.056 (95% CI = 0.053–0.059), which were satisfactory according to our criteria. The final model was used for convergent validity and discriminant validity testing. The standardized regression coefficients of all items were statistically significant, ranging between 0.50 and 0.97, and the ranges of CR and AVE were 0.85–0.92 and 0.50–0.61, respectively (Table 3). The inter-factor correlation coefficients were ≤ 0.80 when examined to test the discriminant validity of the factor construct and the square inter-factor correlation coefficients were lower than the AVE values, which confirmed the discriminant validity of the factors (Table 3).

### 3.3. Criterion Validity

For criterion validity, there was a positive correlation (r = 0.50–0.62, *p* < 0.001) between the Korean ASI and STAI-KYZ (Table 4).

### 3.4. Reliability Analysis

The values of Cronbach’s alpha ranged between 0.71 and 0.88 for the dimensions used in the instrument, as shown by the reliability analysis for the internal consistency of the Korean ASI (Table 4).

## 4. Discussion

Our study aimed to test the validity and reliability of the Korean ASI, which is an instrument used to measure anesthesia surrendering. The tool provides essential data on patients’ anesthesia surrendering experience to medical personnel, to guide the selection of the appropriate intervention to be administered. The validity of an instrument cannot be adequately verified using a single type of validity analysis. Validation studies need to present significant results from multiple analyses to recommend the use of a given instrument in clinical settings [28]. Accordingly, the present study used various methods to analyze three types of validity: content, construct, and criterion. EFA, item analysis, CFA, and criterion validity analysis were performed to verify the construct validity of the Korean ASI. The EFA and CFA were conducted using random samples from the collected data, thereby confirming the measurement equivalence of the instrument. This was necessary for providing robust evidence for the validity of the Korean ASI [16]. In contrast to the validity of the original ASI, which was proven by only EFA, the present study also tested the discriminant and convergent validity of the Korean ASI by CFA and item analysis. Based on the EFA results, four dimensions and a total of 26 items were obtained for the Korean ASI. Since the categorization of the dimensions in the Korean ASI was similar to the ASI developed by Liebenhagen and colleagues [16], our results confirmed that anesthesia surrendering could be measured using the four dimensions of preparation by avoidance, control, preparation by understanding, and acceptance in Korean patients too. However, the item “I looked around the operating theater” in the third dimension had a commonality of 0.10 in the EFA results and it was excluded from the Korean ASI. This item also showed the lowest validity on content validity testing by an expert panel, and it is believed that this may have been due to the layout of the operating rooms in Korea. This is what the waiting time inside the operating room until anesthesia is insufficient for patients to examine the operating theater. It is believed that the patients were unable to afford to slowly look around the operating room. CFA was performed to ensure measurement equivalence of the Korean ASI and the results showed good fitness for the factor structural model of the Korean ASI, which confirmed its construct validity. An item analysis of the factor structure confirmed by EFA was performed to improve content validity and the preliminary convergent validity and discriminant validity were confirmed. Subsequently, factor structure, convergent validity, and discriminant validity were reconfirmed by CFA. In the two rounds of content validity testing, all items met the criteria presented in the previous studies, except the item “I looked around the operating theater” that was found to be unsuitable for Korean patients.

This study also tested the criterion validity of the Korean ASI. Criterion validity should be tested against a gold standard with the same construct as the translated instrument, but no such instrument was available. Previous studies [9,10] have reported that higher anxiety and fear regarding anesthesia result in unfavorable anesthesia outcomes. Therefore, the correlation between the Korean ASI and STAI-KYZ was determined. A previous validity assessment using correlation coefficients [33] determined that correlation coefficients ranging between 0.60 and 0.80 indicate “high validity” and coefficients ranging between 0.80 and 1.0 indicate “very high validity”. Accordingly, the Korean ASI was found to be a valid instrument for measuring anesthesia surrendering in Korean patients. Finally, the internal consistency of the Korean ASI, as measured by the Cronbach’s alpha, ranged between 0.71 and 0.88 in our study. Since the reliability of the ASI at the time of development had a Cronbach’s alpha of 0.76, the Korean ASI was also confirmed to be an instrument with high reliability.

However, our study had certain limitations. A validation test of the ASI requires the use of a gold standard scale that has been validated. For this study, an anesthesia surrendering scale for a concurrent validity test could not be used. Thus, the State-Trait Anxiety Inventory (STAI) scale was used as an alternative. Nevertheless, our analysis is meaningful because this is the first time a study has verified the reliability and validity of a Korean ASI, and the tool is expected to be used for anesthetic nursing care in Korea.

## 5. Conclusions

We translated the ASI developed by Liebenhagen and colleagues for use for Korean patients and tested its validity and reliability so that it would be used to determine appropriate interventions for anesthesia surrendering. The study findings confirmed satisfactory reliability and validity for the four dimensions and 26 items contained in the Korean ASI. This supports its use as an anesthesia surrendering assessment tool in Korean healthcare settings. However, sociocultural factors such as attitudes toward healthcare providers, healthcare under a single payer, and expectations from surgery are expected to affect anesthesia surrender. Additionally, anesthesia surrendering may vary among adults, children, and adolescents. Thus, the development of instruments for measuring anesthesia surrendering in the various demographic groups is also required.

## Figures and Tables

**Table 1:** The item analysis (N = 306).

Items	Mean	Standard deviation	Skewness	Kurtosis
**Factor 1. Preparation by avoidance**				
I tried to avoid thoughts about the anesthesia induction	2.17	0.97	0.14	−1.18
I tried to avoid thoughts about the intraoperative environment	2.28	0.92	−0.21	−1.22
I tried to ignore my emotions	2.28	0.9	−0.05	−0.98
I tried to avoid thoughts about what was happening to me	2.47	0.93	−0.21	−0.89
I tried to control my emotions	2.68	0.9	−0.5	−0.45
I tried to avoid thoughts about what I sensed inside my body	2.43	0.89	−0.2	−0.8
I prepared for postoperative discomfort	2.97	0.91	−0.8	−0.02
**Factor 2. Control**				
I tried to stay awake	2.14	0.91	0.43	−0.95
I tried to keep my eyes open	2.07	0.92	0.5	−0.93
I tried to maintain control	2.35	0.98	−0.01	−1.09
I felt I was being suffocated	1.91	0.97	0.68	−0.66
I was afraid of waking up during surgery	2.21	0.91	0.32	−1.29
I felt defenseless (before induction)	2.46	0.94	−0.07	−1.18
I was afraid of not regaining consciousness	2.4	0.99	0.08	−1.29
**Factor 3. Preparation by understanding**				
I tried to pose as many questions as possible to the anesthesia provider	1.93	0.84	0.62	−0.22
I tried to understand the technical objects	2.09	0.13	1.05	1.23
I tried to understand what was taking place around me	2.4	0.92	−0.11	−0.91
I tried to envision the anesthesia	2.04	0.88	0.37	−0.75
I observed the work of the anesthesia provider	2.19	0.92	0.12	−1.03
I tried to joke	1.7	1	1.01	1.92
**Factor 4. Acceptance**				
I felt that I could surrender	2.71	0.88	−0.48	−0.4
I made eye contact with the anesthesia provider	2.2	0.97	0.1	−1.17
I followed the AP’s instructions	3.27	0.76	−1.1	1.42
I experienced being personally received by the anesthesia provider	2.49	0.87	−0.23	−0.68
I breathed deeply into the anesthesia mask	3.08	0.97	1.92	1.41
I felt defenseless (at induction)	2.78	0.92	−0.47	−0.53

**Table 2:** Exploratory factor analysis of anesthesia surrendering instrument (N = 306).

Factors and Items	Factor Loading			
	1	2	3	4
**Factor 1. Preparation by Avoidance**				
I tried to avoid thoughts about the anesthesia induction	0.66	0.28	0.15	0.13
I tried to avoid thoughts about the intraoperative environment	0.88	0.16	0.04	0.19
I tried to ignore my emotions	0.81	0.06	0.12	0.17
I tried to avoid thoughts about what was happening to me	0.75	0.17	0.09	0.05
I tried to control my emotions	0.7	0.15	0.19	0.11
I tried to avoid thoughts about what I sensed inside my body	0.64	0.17	0.13	−0.03
I prepared for postoperative discomfort	0.9	0.13	−0.07	0.1
**Factor 2. Control**				
I tried to stay awake	0.15	0.91	0.14	−0.01
I tried to keep my eyes open	0.14	0.83	0.14	0
I tried to maintain control	0.26	0.65	0.1	0.11
I felt I was being suffocated	0.16	0.78	0.13	−0.14
I was afraid of waking up during surgery	0.22	0.66	0.19	−0.1
I felt defenseless (before induction)	0.24	0.73	0.17	0.1
I was afraid of not regaining consciousness	0.1	0.71	0.16	0.02
**Factor 3. Preparation by understanding**				
I tried to pose as many questions as possible to the anesthesia provider	0.32	0.17	0.78	0.14
I tried to understand the technical objects	0.1	0.06	0.81	0.11
I tried to understand what was taking place around me	0.21	0.18	0.75	0.11
I tried to envision the anesthesia	0.17	0.2	0.73	0.21
I observed the work of the anesthesia provider	0.24	0.06	0.7	0.15
I tried to joke	0.17	0.04	0.68	0.27
**Factor 4. Acceptance**				
I felt that I could surrender	−0.07	0.09	−0.14	0.74
I made eye contact with the anesthesia provider	−0.08	0	0.13	0.74
I followed the AP’s instructions	−0.07	0.03	−0.01	0.61
I experienced being personally received by the anesthesia provider	0.23	−0.07	0.2	0.69
I breathed deeply into the anesthesia mask	0.3	−0.05	0.18	0.66
I felt defenseless (at induction)	0.28	0.15	0.13	0.62
Eigenvalue	4.9	4.37	3.7	3.11
% of variance	18.84	16.8	14.23	11.96
% of cumulative	18.84	35.64	49.87	61.83

**Table 3:** Confirmatory factor analysis of anesthesia surrendering instrument (N = 306).

Items	Factor loading	SE	*p*	CR	AVE
**Factor 1. Preparation by Avoidance**				0.91	0.6
I tried to avoid thoughts about the anesthesia induction	0.8	0.02	<0.001		
I tried to avoid thoughts about the intraoperative environment	0.86	0.02	<0.001		
I tried to ignore my emotions	0.82	0.02	<0.001		
I tried to avoid thoughts about what was happening to me	0.84	0.02	<0.001		
I tried to control my emotions	0.82	0.02	<0.001		
I tried to avoid thoughts about what I sensed inside my body	0.72	0.03	<0.001		
I prepared for postoperative discomfort	0.5	0.05	<0.001		
**Factor 2. Control**				0.92	0.61
I tried to stay awake	0.95	0.01	<0.001		
I tried to keep my eyes open	0.97	0.01	<0.001		
I tried to maintain control	0.74	0.02	<0.001		
I felt I was being suffocated	0.64	0.04	<0.001		
I was afraid of waking up during surgery	0.74	0.03	<0.001		
I felt defenseless (before induction)	0.66	0.04	<0.001		
I was afraid of not regaining consciousness	0.72	0.03	<0.001		
**Factor 3. Preparation by understanding**				0.88	0.55
I tried to pose as many questions as possible to the anesthesia provider	0.78	0.03	<0.001		
I tried to understand the technical objects	0.68	0.03	<0.001		
I tried to understand what was taking place around me	0.56	0.04	<0.001		
I tried to envision the anesthesia	0.81	0.03	<0.001		
I observed the work of the anesthesia provider	0.78	0.03	<0.001		
I tried to joke	0.79	0.03	<0.001		
**Factor 4. Acceptance**				0.85	0.5
I felt that I could surrender	0.76	0.08	<0.001		
I made eye contact with the anesthesia provider	0.82	0.06	<0.001		
I followed the AP’s instructions	0.54	0.06	<0.001		
I experienced being personally received by the anesthesia provider	0.53	0.06	<0.001		
I breathed deeply into the anesthesia mask	0.82	0.06	<0.001		
I felt defenseless (at induction)	0.7	0.07	<0.001		

SE—Standard error, CR—Construct reliability, AVE—Average variance extracted.

**Table 4:** Correlation among sub-factors of anesthesia surrendering instrument and State-Trait Anxiety Inventory (N = 306).

Variables	Factor 1	Factor 2	Factor 3	Factor 4
Factor 1_preparation by avoidance	**0.6**	-	-	-
(Cronbach’s alpha = 0.88)				
Factor 2_control	0.55 *	**0.61**	-	-
(Cronbach’s alpha = 0.82)				
Factor 3_preparation by understanding	0.54 *	0.57 *	**0.55**	-
(Cronbach’s alpha = 0.80)				
Factor 4_acceptance	0.51 *	0.52 *	0.59 *	**0.5**
(Cronbach’s alpha = 0.71)				
State–Trait Anxiety Inventory	0.62 *	0.62 *	0.50 *	0.51 *
(Cronbach’s alpha = 0.87)				

The oblique bolded section: discriminant validity, The non-bolded section: correlation, * *p* < 0.001.
